# Characterization of Clinically Evaluated Small-Molecule Inhibitors of PD-L1 for Immunotherapy

**DOI:** 10.1021/acsmedchemlett.5c00245

**Published:** 2025-06-09

**Authors:** Alicja Slota, Katarzyna Golebiowska-Mendroch, Justyna Kocik-Krol, Bogdan Musielak, Malgorzata Stec, Kazimierz Weglarczyk, Maciej Siedlar, Lukasz Skalniak, Jacek Plewka, Katarzyna Magiera-Mularz

**Affiliations:** [a] 37799 Jagiellonian University, Faculty of Chemistry, Department of Organic Chemistry, Gronostajowa 2, 30-387 Krakow, Poland; [b] 37799 Jagiellonian University, Doctoral School of Exact and Natural Sciences, prof. St. Lojasiewicza 11, 30-348 Krakow, Poland; [c] 49573 Jagiellonian University Medical College, Department of Clinical Immunology, Institute of Pediatrics, Wielicka 265, 30-663 Krakow, Poland

**Keywords:** cancer immunotherapy, small-molecule inhibitors, PD-L1, clinical trials

## Abstract

Cancer immunotherapy aims to employ the immune system to target cancer cells. The PD-1/PD-L1 axis is a critical immune checkpoint that tumors exploit to evade immune surveillance. In this study, we characterized three small-molecule PD-L1 inhibitors, Evixapodlin, MAX-10181, and INCB086550, currently undergoing clinical trials for cancers such as non-small cell lung cancer, renal cell carcinoma, urothelial carcinoma, hepatocellular carcinoma, and melanoma. Using the homogeneous time resolved fluorescence assay, we confirmed that each compound potently disrupts human PD-1/PD-L1 binding with IC_50_ values in the nanomolar range. PD-L1 oligomerization upon inhibitor binding was demonstrated through NMR analysis and confirmed by X-ray crystallography, which finally elucidated the binding interactions that stabilize these inhibitors at the PD-L1 interface. Cellular assays revealed dose-dependent T-cell activation, demonstrating the immunomodulatory potential of each compound and its cytotoxicity profiles. These findings underscore the promise of small-molecule PD-L1 inhibitors as viable alternatives to antibody-based therapies in cancer immunotherapy.

## Graphical abstract

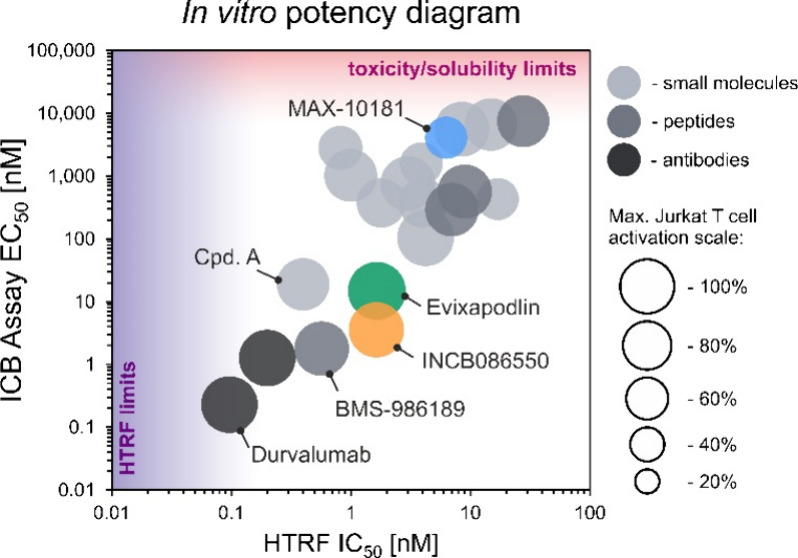

The immune system plays a crucial role in detecting and eliminating cancer cells, yet many cancers develop mechanisms to evade immune responses. A common tactic involves altering immune checkpoints, such as PD-1, which, when engaged by its ligand PD-L1 on tumor cells, suppresses immune activity and reduces the immune system’s ability to target cancer cells.^1^ Therapeutic antibodies targeting PD-1 or PD-L1 have proven effective across several types of cancer by blocking this inhibitory pathway, thus, allowing the immune system to recognize and destroy cancer cells.^2^ The FDA has approved multiple PD-1- or PD-L1-blocking antibodies for clinical use, highlighting their therapeutic success.^3^ In preclinical models, inhibiting PD-1/PD-L1 interaction enhances T-cell responses, stimulates cytokine release, boosts T cell-mediated tumor cell killing, and increases CD8+ T-cell infiltration into tumors.^4^ While antibody-based therapies offer significant benefits, small-molecule inhibitors (SMIs) for PD-1/PD-L1 bring unique advantages, including shorter half-lives for flexible dosing schedules that can reduce immune-related side effects. Additionally, small molecules may have better tissue penetration and the possibility of oral administration, making them promising options as standalone treatments or in combination with other targeted therapies. This, combined with significantly lower production costs, makes SMIs a viable alternative to monoclonal antibody-based cancer therapies currently available.^5^ Despite the promise of small-molecule PD-L1 inhibitors, the FDA has not yet approved any for cancer treatment. Currently, the PD-1/PD-L1 checkpoint blockade in oncology relies on monoclonal antibodies, but small molecules targeting immune checkpoints, including PD-L1, are under active investigation in clinical trials. Notable examples include INCB086550, in Phase II trials for various cancers, and INCB099280, also in Phase II trials for multiple cancers.^6,7^

Out of ten small-molecule inhibitors targeting PD-L1 currently in clinical trials, only four have their chemical structures disclosed: Evixapodlin (also known as GS-4224; PD-1/PD-L1-IN 7; CAS No.: 2374856-75-2) developed by Gilead Sciences,^8^ MAX-1018 (also known as PD-1/PD-L1-IN-30; CAS No.: 2171558-14-6) developed by Maxinovel Pty., Ltd.,^9^ INCB086550 (also known as PD-1/PD-L1-IN-8; CAS No.: 2230911-59-6) developed by Incyte Corporation,^6^ and IMMH-010 (also known as YPD-30; CAS No.: 2541982-47-0) developed by Tianjin Chasesun Pharmaceutical Co., Ltd.^10^ which is a prodrug. Structurally, they represent various classes: MAX-1018 represents classical BMS-like compounds with a core biphenyl structure fused with a 1,4-dioxane ring on one side and on the other linked via rigidified double-bonded linker with a distal benzene ring substituted with a trifluoromethyl group and α-methylserine solubilizer (Figure 1). Evixapodlin is a member of C2-symmetric PD-L1 inhibitors with a rigid modified quaterphenyl core where the central biphenyl is decorated with chlorine atoms, while the distal rings are pyrazines both substituted with methoxyl groups and (aminomethyl)­pyrrolidin-2-one solubilizers. INCB086550 is a C2-asymmetric PD-L1 inhibitor with a central biphenyl substituted with methyl groups. One side has a 1,7-naphthyridine connected via an amine linker and pyrrolidinol solubilizer, while the other side has a 1,3-benzoxazole moiety substituted with nitrile group and pyrrolidine-3-carboxylic acid solubilizer. Both INCB086550 and Evixapodlin are significantly larger than MAX-1018 at approximately 700 and 500 Da, respectively, and significantly exceed Lipinski’s rule of five. All of these compounds have relatively high theoretical octanol–water partition coefficients (LogP), suggesting possible low bioavailability due to high lipophilicity. However, our previous study showed a low correlation between theoretical and experimental solubility of compounds, indicating the need for experimental determination of LogP rather than relying on calculations in the drug design process.^11^ Among those compounds, they all have acceptable number of hydrogen bond acceptors (<12) and donors (<7).

**Figure 1:**
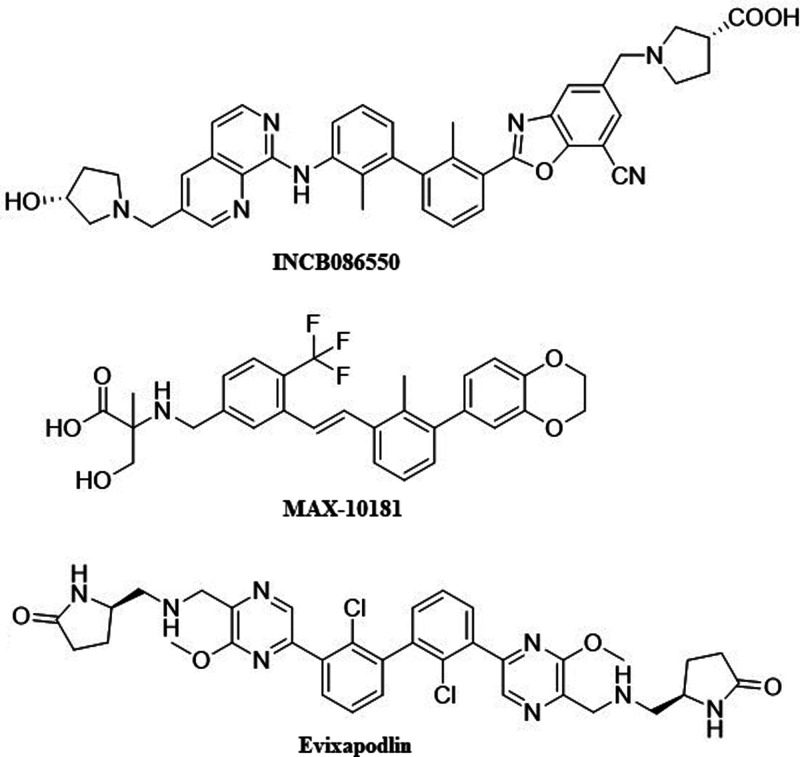
Chemical structures of PD-L1 small-molecule inhibitors.

To assess their potency in disrupting the human PD-1/PD-L1 complex, we purchased the aforementioned compounds, dissolved them in DMSO, and tested them using the gold-standard homogeneous time resolved fluorescence (HTRF) assay for PD-L1 inhibitors. The resulting half-maximal inhibitory concentration (IC_50_) values were in the low nanomolar range, close to those reported in the literature, proving their high efficacy.

**1:** Molecular Weight, Lipophilicity, and Potency in Disrupting PD-1/PD-L1 for Anti-PD-L1 Small-Molecule Inhibitors That Are Currently in Clinical Trials

Name	MW [Da]	LogP	IC_50_ [nM] HTRF	IC_50_ [nM] literature
INCB086550	693	6.8	2.59 ± 0.09	3.1 ± 1.2 ^6^
MAX-10181	527	5.5	6.88 ± 0.12	18 ^12^
Evixapodlin	691	4.3	1.74 ± 0.15	0.21 ^13^

The direct binding of compounds Evixapodlin, MAX-10181, and INCB086550 to human PD-L1 was confirmed using a ^1^H NMR method (Figure 2A). In this assay, the addition of mentioned compounds induced a characteristic oligomerization of PD-L1, previously observed for biphenyl compounds,^14–16^ as evidenced by peak broadening in the aliphatic region of the ^1^H NMR spectrum compared to the spectrum of apo PD-L1. This NMR approach provides unambiguous results, making it an excellent orthogonal validation tool for HTRF, as it does not require fluorescence modifications and thus eliminates false-positive signals from autofluorescent compounds or quenchers.

**Figure 2:**
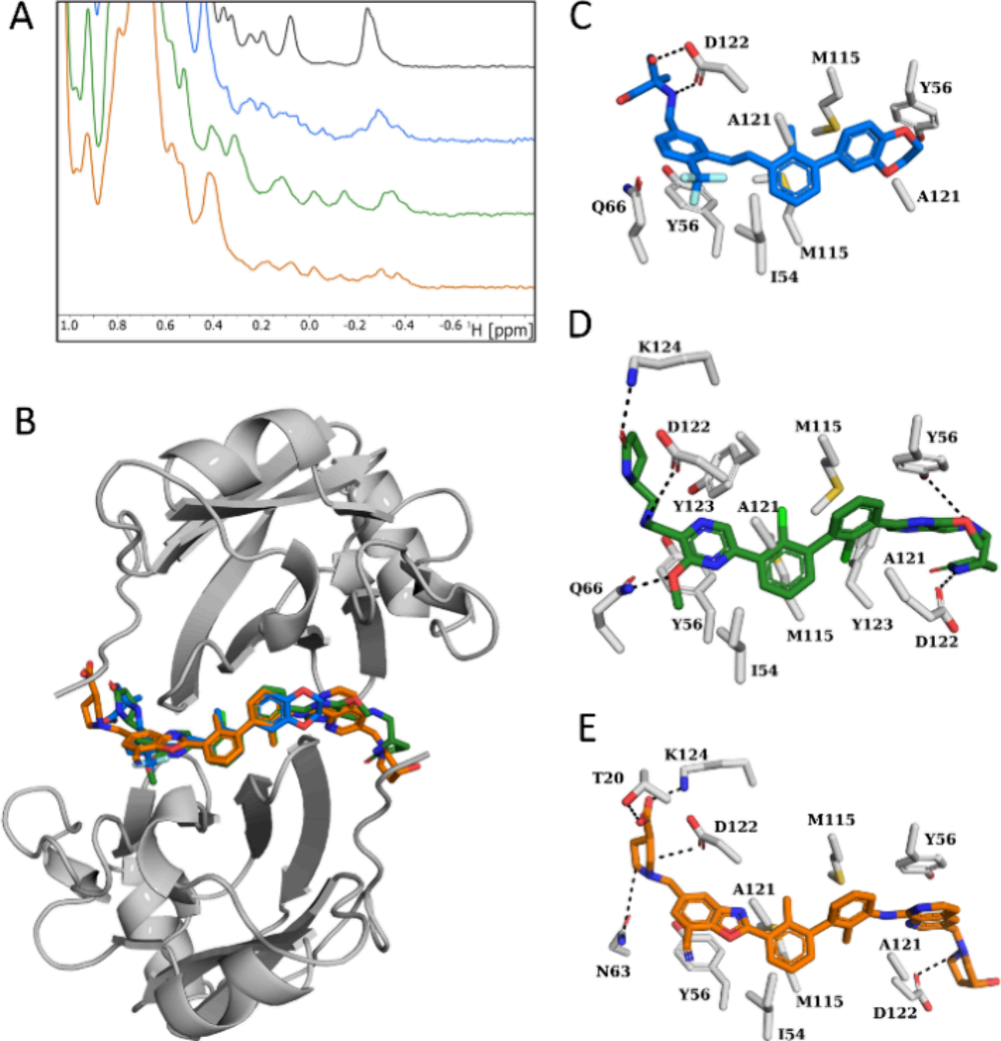
SMIs induce PD-L1 oligomerization confirmed by NMR measurements and the X-ray structure. (A) The aliphatic part of the ^1^H NMR spectrum of PD-L1 protein (gray) and with inhibitors: MAX-10181 (blue), Evixapodlin (green), and INCB086550 (orange), in the molar ratio protein to inhibitor 1:3. (B–E) Crystal X-ray structures of the PD-L1/inhibitor complexes. (B) Overall view of the PD-L1/inhibitor binding interfaces. (C) Detailed interactions of MAX-10181 at the binding interface (PDB: 9HRT). (D) Detailed interactions of Evixapodlin at the binding interface (PDB: 9I0U). Detailed interactions of INCB086550 at the binding interface (PDB: 9I0W).

The small-molecule PD-L1 inhibitors analyzed here were also subjected to a series of cellular assays to evaluate their bioactivity and toxicity. First, the PD-1/PD-L1 immune response checkpoint blockade (ICB) assay was performed, which requires the use of a co-culture of two cell lines that interact with each other via PD-1 interaction with PD-L1. The first cell line consisted of artificial antigen-presenting cells (aAPCs), which were CHO/TRCAct/PD-L1 cells, while the second cell line consisted of Jurkat effector cells (Jurkat-ECs). The co-culture of these two cell lines could lead to the activation of Jurkat-ECs by the T-cell receptor (TCR), however, only when PD-1 interaction with PD-L1 is inhibited. Thus, the presence of PD-L1 inhibitors in the system can lead to the restoration of Jurkat-ECs activation and its determination by luminescence measurement (Figure 3A).

**Figure 3:**
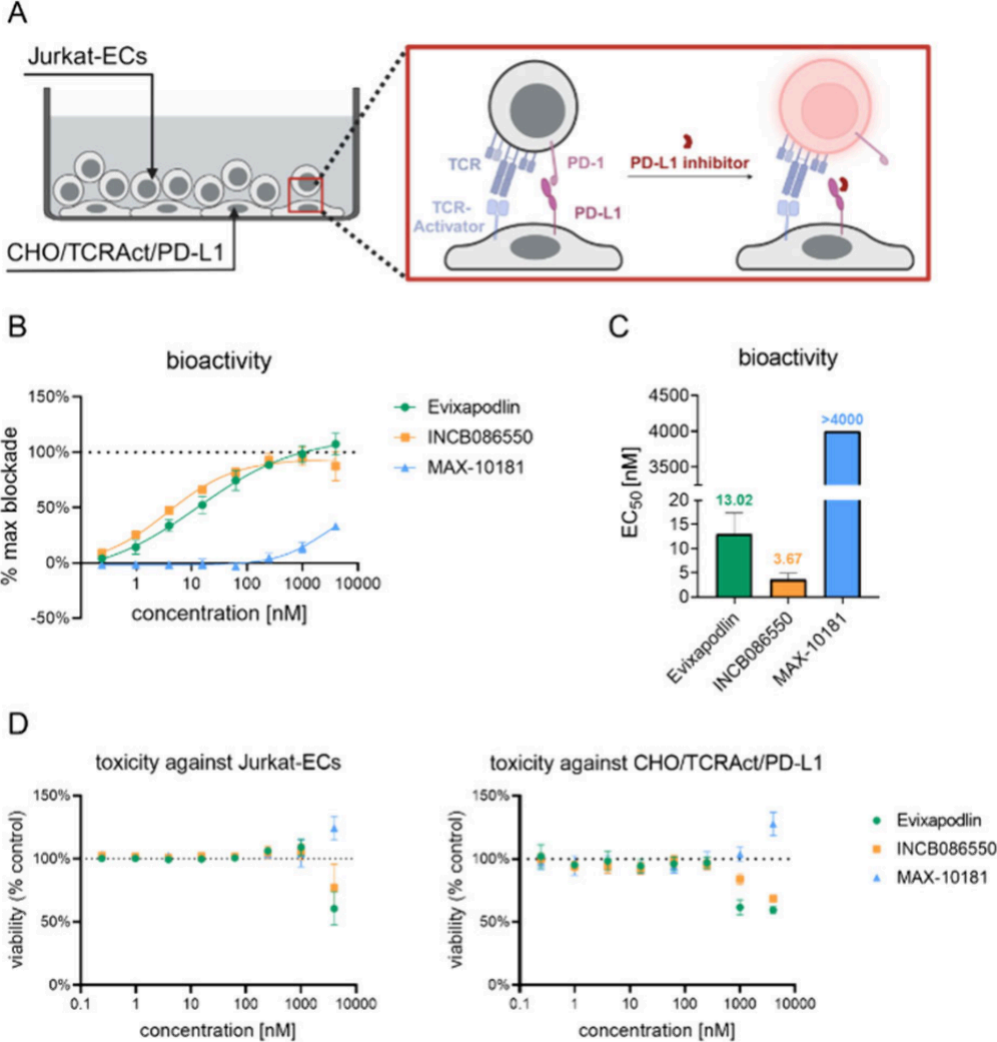
Bioactivity and toxicity of small-molecule PD-1/PD-L1 inhibitors. (A) Schematic representation of the principle of cellular PD-1/PD-L1 immune checkpoint blockade assay. (B) Dose-dependent activation of Jurkat-ECs. Measured luminescence signal is shown as % of maximal blockade, where 100% represents activation of Jurkat-ECs after incubation with a positive control, an antibody (durvalumab 5 μg/mL), and 0% represents activation of these cells after incubation with a negative control (DMSO). Data points represent mean ± SD values from 5 independent experiments, each in duplicate. (C) EC_50_ of the bioactivity of the compounds. (D) Cytotoxicity of Evixapodlin, INCB086550, and MAX-10181 against Jurkat-ECs cells (left) and CHO/TCRAct/PD-L1 cells (right panel). Data points represent mean ± SD values from five (left panel) or four (right panel) independent experiments, each in duplicate.

As a positive control in this experiment, we used durvalumab, which activates effector cells with an EC_50_ value of 0.2 ± 0.06 nM.^16^ All of the compounds tested here caused the release of TCR signaling but at significantly higher concentrations than durvalumab. Application of INCB086550 and Evixapodlin resulted in a dose-dependent restoration of Jurkat-ECs activation with EC_50_ values of 3.67 ± 1.21 nM and 13.02 ± 4.33 nM, respectively (Figure 3B, C). In the case of compound MAX-10181, no activation plateau was observed for the concentration range tested (Figure 3B, C), and the determined EC_50_ value was >4000 nM. Subsequently, the toxicity of these compounds was checked against the cell lines used in the ICB method. For this purpose, Jurkat-ECs and CHO/TCRAct/PD-L1 cells were subjected to colorimetric cell viability tests in which toxicity was determined (Figure 3D). After 48 h, a decrease in effector cell viability was observed at concentrations above 1 μM, while only compound concentrations above 0.25 μM were toxic to CHO/TCRAct/PD-L1 cells (Figure 3D).

To evaluate the compounds’ ability to trigger T-cell response, the T-cell activation (TCA) assay was performed. CHO/TCRAct/PD-L1 cells when contacted with Peripheral Blood Mononuclear Cells (PBMCs, consisting of T, B, Natural Killer cells, and monocytes), suppress the activity of the immune system through the PD-1/PD-L1 interaction. Thus, the presence of immune checkpoint inhibitors should lead to the immune cells reactivation. In the assay, we evaluated activation of CD4+ and CD8+ T cells by measuring the expression of early (CD69), intermediate (CD25 and HLA-DR), and late (PD-1) activation/exhaustion markers (Figure 4, Figure S2, Figure S3). As presented in our previous studies, PD-1/PD-L1 blockers introduced in this assay, enhance mainly PD-1 expression on T cells, while having a significantly reduced impact on the expression of other markers.^16–19^

**Figure 4:**
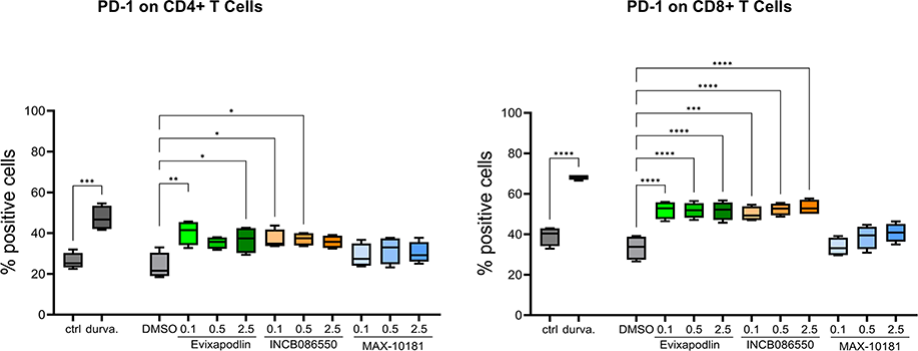
Examination of Evixapodlin, INCB086550, and MAX-10181 ability to reactivate primary human CD4+ and CD8+ lymphocytes T in the co-culture with PD-L1 presenting cells. The expression of PD-1 as a late T-cell activation and exhaustion marker was assessed after 48 h of co-culture of PBMCs with CHO/TCRAct/PD-L1 in the presence of tested inhibitors at the final concentrations: 0.1, 0.5, 2.5 μM, and durvalumab (durva.) at the final concentration 5 μg/mL. On the graphs, the fractions of PD-1 expressing cells among CD4+ or CD8+ (% positive cells) are shown as calculated data from four independent experiments involving PBMCs from four healthy blood donors. Statistical significance was analyzed in GraphPad using one-way ANOVA, followed by the posthoc Tukey test * *p* < 0.05, ** *p* < 0.01, ***, *p* < 0.000, **** *p* < 0.00001.

In this setup, Evixapodlin, INCB086550, and durvalumab significantly increase the number of PD-1-positive CD4+ and CD8+ cells, with the latter being the most powerful effectors in the anticancer immune response due to their cytotoxicity (18) (Figure 4). This effect was not observed in the experiment variant with CHO/TCRAct cells co-cultured with PBMCs indicating the specificity of the activity of the tested inhibitors toward PD-L1 (Figure S3).

To explore the interaction pattern of inhibitors with the PD-L1 protein at a molecular level, we cocrystallized and solved the structures of PD-L1 in complex with Evixapodlin, INCB086550, and MAX-10181 at resolutions of 1.5, 2.1, and 2.3 Å, respectively (Figure 2B, Table S1). All asymmetric units of the complexes contain two PD-L1 chains arranged into a homodimer with the inhibitor found at the interface of the dimer, similar to repeatedly reported by us and others.^14,15,19^ Consistent with this structural mode of dimerization, Koblish et al.^6^ reported that INCB086550 induces PD-L1 dimerization, which is further linked to its internalization and nuclear translocation leading to reduced surface expression and enhanced T cell activation.

The obtained electron density map of all compounds is well-defined, allowing for detailed characterization of protein-inhibitor interactions and structure–activity relationship analysis (Figure 2C–E). The strong stabilization of the structures is provided through classical π–π stacking between the pyridine rings of Evixapodlin and _A_,_B_Tyr56, while the same interaction was found for 1,7-naphthyridine and 1,3-benzoxazole moieties of INCB086550. Contrary, unsymmetrical and less elongated MAX-10181 provides only one π–π stacking interaction between the (trifluoromethyl)­benzene ring and _B_Tyr56. Numerous other hydrophobic interactions can be found in each complex, which are mostly created with the central part of the inhibitors located in the hydrophobic channel, formed between two molecules of PD-L1. The nonpolar bonds common to all structures include interactions with _B_Ile54, _A_,_B_Met115, and _A_,_B_Ala121. The PD-L1/MAX-10181 complex is additionally stabilized by π–alkyl interactions of _A_Tyr56 with the 1,4-dioxane moiety and _B_Tyr56 with the trifluoromethyl group. However, due to lack of halogen groups on biphenyl, it lacks stabilizing interactions with, e.g., _A_Asp108. Moreover, alkyl interactions between _A_,_B_Tyr123 and pyrrolidinone solubilizers of Evixapodlin provide a more rigid arrangement of the distal parts of the compound to the PD-L1 surface. The presence of the halogen elements in the structures of Evixapodlin and MAX-10181 provides additional halogen bonds with _A_Asp122 and _B_Gln66, respectively, although with minor contributions. In turn, the area of the polar contacts is mainly located at the outside of the hydrophobic tunnel and is definitely poor for MAX-10181 including two hydrogen bonds between _A_Asp122 and the α-methylserine moiety. Contrary, structures of C2-symmetrical compounds extended by introducing solubilizer groups provide an extensive network of polar interactions. The Evixapodlin/PD-L1 structure is stabilized by hydrogen bonds with _B_Tyr56, _B_Gln66, _A_,_B_Asp122, and _A_Lys124. Similar polar interactions are observed in the INCB086550/PD-L1 structure, except for contact with _B_Gln66. Interestingly, the pyrrolidine-3-carboxylic acid moiety of INCB086550 adopts a bent conformation, pointing toward _A_Thr20 and forming a hydrogen bond between solubilizer and threonine carboxylic groups. This arrangement maintains an appropriate distance to preserve the standard polar interaction with _A_Lys124, while positioning this distal moiety in close proximity to _B_Asn63 and ensuring further stabilization by a carbon–hydrogen bond. This interaction mimics those previously observed by us in the structures of macrocyclic peptide/PD-L1 complexes, which represent the most potent non-antibody-based inhibitors of the PD-1/PD-L1 pathway, so far (Figure S4). This feature can explain the high potential of INCB086550 as demonstrated here. Further, distal parts of both Evixapodlin and INCB086550 are also mainly water-exposed, which significantly influences their solubility in aqueous solutions.

In conclusion, our study provides strong evidence of the potential of small-molecule PD-L1 inhibitors as alternatives to antibody-based therapies in cancer immunotherapy. The detailed biochemical, structural, and cellular characterization of INCB086550, Evixapodlin, and MAX-10181 lays the groundwork for future optimization and research, particularly highlighting the effects of INCB086550 and Evixapodlin. INCB086550 emerged as the most potent compound in our assays. It is noteworthy for its performance, second only to the macrocyclic peptide BMS986189 from Bristol Myers Squibb (BMS).^18–21^ INCB086550 has been extensively studied, with clinical trials starting in 2018 (NCT03762447), demonstrating its safety and efficacy in patients with solid tumors. *In vitro* studies showed no adverse effects on pleural effusion fluid cells, while *in vivo* studies indicated a reduction of PD-L1 on tumor cell surfaces and inhibition of tumor growth in mouse model.^6^ This compound is also part of several ongoing clinical trials assessing its pharmacokinetics and efficacy in various cancers, although results are pending (NCT05101369, NCT04674748, and NCT04629339).

Evixapodlin has shown promising results in both *in vitro* and *in vivo* studies, increasing the level of IFN-γ and Grazyme B production in T cells and reducing PD-L1 levels on tumor cells. Its phase I clinical trial (NCT04049617) demonstrated safety and tolerability in patients with advanced solid tumors, supporting its potential as an anti-PD-L1 agent.^8^

No data on *in vitro* and *in vivo* studies for MAX-10181 have been published to date; however, it is currently in phase I trials in Australia and China (NCT04122339 and NCT05196360). In our study, MAX-10181 was less effective than INCB086550 and Evixapodlin, likely due to its classification as a classical BMS-like compound such as BMS-1166,^22^ not C2-symmetrical, inhibitor such as compound A.^23^

As the field of cancer immunotherapy evolves, small-molecule inhibitors are expected to play a significant role, broadening treatment options and providing more accessible therapies for patients with various cancers.

## Supplementary Material



## Notes

Sources of inhibitors; experimental protocols for protein expression, purification, crystallization, and structure determination; procedures for HTRF, NMR binding, and cell-based assays; as well as supplementary figures and a table with X-ray structural data (PDF)

## Notes

Coordinates and structure factors were deposited in the Protein Data Bank with accession numbers: 9HRT (PD-L1/ MAX-1018), 9I0U (PD-L1/Evixapodlin) and 9I0W (PD-L1/ INCB086550). Authors will release the atomic coordinates and experimental data upon article publication.

## Abbreviations

aAPCs: artificial antigen-presenting cells
BMS: Bristol-Myers Squibb
DMSO: dimethyl sulfoxide
EC_50_: half maximal effective concentration
ECs: effector cells
FDA: Food and Drug Administration
HTRF: homogeneous time resolved fluorescence
IC_50_: half maximal inhibitory concentration
ICB assay: immune checkpoint blockade assay
IFN-γ: interferon-gamma
NMR: nuclear magnetic resonance
PBMCs: peripheral blood mononuclear cells
PD-1: programmed death receptor 1
PD-L1: programmed death-ligand 1
PBD: Protein Data Bank
SMIs: small-molecule inhibitors
TCA: T-cell activation
TCR: T-cell receptor
TCRAct: TCRActivator.
