# Supplementary material for: Characterization of Clinically Evaluated Small-Molecule Inhibitors of PD-L1 for Immunotherapy
Source: ACS Med Chem Lett. 2025 Jun 9;16(7):1359–64. doi: 10.1021/acsmedchemlett.5c00245 (PMC12257390; doi:10.1021/acsmedchemlett.5c00245)
Supplement: Supplementary file 1 [file ml5c00245_si_001.pdf]

Supporting Information

Characterization of clinically evaluated small-molecule inhibitors of PD-L1 for immunotherapy.

Alicja Slota[a,b], Katarzyna Golebiowska-Mendroch[a,b], Justyna Kocik-Krol[a], Bogdan Musielak[a], Malgorzata Stec[c], Kazimierz Weglarczyk[c], Maciej Siedlar[c], Lukasz Skalniak[a], Jacek Plewka[a]*, Katarzyna Magiera-Mularz[a]*

[a] Jagiellonian University, Faculty of Chemistry, Department of Organic Chemistry, Gronostajowa 2, 30-387 Krakow, Poland
[b] Jagiellonian University, Doctoral School of Exact and Natural Sciences, prof. St. Lojasiewicza 11, 30-348 Krakow, Poland
[c] Jagiellonian University Medical College, Department of Clinical Immunology, Institute of Pediatrics, Wielicka 265, Krakow, 30-663, Poland

* corresponding authors contributed equally
E-mail addresses: jacek.plewka@uj.edu.pl, k.magiera@uj.edu.pl

Supporting Information

Table of contents:

Experimental Procedures and Materials page 3

Supplementary Figures and Tables page 7

Author Contributions page 11

Experimental Procedures and Materials

Small-molecule inhibitors source

Evixapodlin, MAX-10181, and INCB086550 compounds were purchased from MedChemExpress and dissolved in DMSO to prepare 50 mM stock solutions. The vendor assured a purity of >98% for the batches, as determined by HPLC and LC-MS.

Homogeneous Time-Resolved Fluorescence

The assay was performed using the certified kit (Revvity) following the manufacturer's instructions. The experiments were conducted on 96-well white plates in a final volume of 20 µl. The measurements were performed in two separate dilution series of the tested compounds with excitation at 320 nm and measuring fluorescence intensity at 620 nm and 665 nm wavelengths using Tecan Spark 20M. The backgrounds of obtained data were subtracted from the negative control, normalised to the positive control and averaged. Errors were calculated using sample standard deviations from these two measurements. To determine the half maximal inhibitory concentration (IC_{50}) a set of measurements of different compound concentrations (ranging from 10^{-7} M to 10^{-12} M) by fitting the results with Hill's equation using Mathematica.

Protein Expression and Purification

Escherichia coli BL21(DE3) cells were transformed with a plasmid encoding human PD-L1, residues 18-134, C-terminal His-tag and plate on Ampicillin LB-agar. The bacteria were grown in LB at 37°C until reaching OD_{600} of 0.6, lowered the temperature to 28°C and induced at 1 mM IPTG, and cultured overnight. Proteins were expressed as inclusion bodies which were harvested by centrifugation. These were washed initially with PBS, followed by two washes in a buffer containing 50 mM Tris pH 8.0, 200 mM NaCl, 10 mM EDTA, 0.5% Triton X-100 and 10 mM β-mercaptoethanol and once again in the buffer above but with no Triton X-100. Each washing step was accompanied by sonication and centrifugation. The washed inclusion bodies were solubilized by stirring overnight at 4°C in a solution of 6 M GdnHCl, 50 mM Tris pH 8.0, 200 mM NaCl, 2 mM EDTA and 10 mM β-mercaptoethanol. A solubilised fraction was clarified by high-speed centrifugation and stored at 4°C. Refolding of the proteins was achieved by dropwise dilution into the refolding buffer containing 0.1 M Tris pH 8.0, 1 M L-Arginine-HCl, 2 mM EDTA, 0.25 mM glutathione oxidized and 0.25 mM glutathione reduced. Afterwards, proteins were dialysed 3 times for 24 hours against a buffer consisting of 10 mM Tris pH 8.0 and 20 mM NaCl. Proteins were purified by Size-Exclusion Chromatography on HiLoad 26/600 Superdex 75 column in the same buffer.

PD-L1 co-crystallization, Determination and Refinement

Purified PD-L1 in 10 mM Tris pH 8.0, 20 mM NaCl buffer was concentrated to 8 mg/ml, mixed with inhibitor at 1:3 molar ratio (protein:compound) and clarified. Commercially available buffer sets were used to screen for crystallisation conditions. Diffraction-quality crystals of PD-L1/Evixapodlin complex were obtained at room temperature from the condition containing 2.0 M ammonium sulfate; 0.1 M sodium acetate pH 4.6, those of PD-L1/MAX-10181 complex from 0.2 M ammonium sulfate; 0.1 M sodium cacodylate; 6.53% w/v PEG 8000 and PD-L1/INCB086550 from condition 0.2 M ammonium sulfate, 0.1 M Bis-Tris pH 5.5, 25% w/v PEG 3350. The crystals were flash-frozen in liquid nitrogen with glycerol as cryoprotection.

The X-ray diffraction data was collected at the PETRA III P11 beamline at DESY (Hamburg, Germany) and ID30A-3 beamline at ESRF (Grenoble, France). The data were indexed and integrated using XDS, scaled using Aimless and processed in CCP4 Cloud. The initial phases were obtained by molecular replacement using Phaser and PDB ID: 5N2F as a model. The model was built using Coot, refinement was performed in Refmac5. Water molecules were added automatically and inspected manually.

Coordinates and structure factors were deposited in the Protein Data Bank with accession numbers: 9HRT (PD-L1/ MAX-1018), 9I0U (PD-L1/Evixapodlin) and 9I0W (PD-L1/ INCB086550).

Cell culture

The experiments used adherent hamster-derived ovarian cancer cells (CHO-K1) with overexpression of TCR activator and human PD-L1 on the cell surface (CHO/TCRAct/PD-L1) (Promega, Madison, WI). Acute T-cell leukaemia (Jurkat T) cells overexpressing PD-1, which had a luciferase reporter gene under the control of Nuclear Factor of Activated T-cells Response Element (NFAT-RE) (PD-L1 Effector Cells, PD-L1 ECs, Promega), were also used. Both cell lines were cultured in 75 cm^2 flat-bottomed bottles (Corning Falcon) in a Galaxy 170 R incubator (Eppendorf) at 37℃, in the presence of 5% carbon dioxide. RPMI-1640 medium with stable Glutami (Biowest) with the addition of 10% Fetal Bovine Serum (FBS, Biowest) and 2 mM L-Glutamine (Biowest) was used. In addition, selection antibiotics - G418 at 250 ug/mL (InvivoGen, San Diego, CA) and Hygromycin B Gold at 50 µg/mL (InvivoGold) - were added to the medium. Cells were maintained in logarithmic growth, while reduction passages or seeding for experiments were performed when cells reached approximately 80% confluence. For adherent cells, the culture medium was removed and the cells were washed twice with phosphate-buffered saline (PBS) without Calcium and Magnesium (VWR). After a short incubation in trypsin with EDTA 0.02% v/v (Biowest), the cells were detached from the bottle. The trypsin was then inactivated with 4 mL of complete culture medium and the cells were centrifuged for 5 minutes at 200 g. For suspension cells, cells were harvested along with the culture medium into 15 mL Falcon tubes (Corning Falcon) and centrifuged at the same parameters as the adherent cells. After centrifugation, pellets of both cell types were resuspended in a fresh culture medium and the cells were then counted in a Bürker chamber. Cell lines were in culture for no longer than 30 days, after which the new banks were thawed. Mycoplasma contamination of the cell lines was routinely checked using the MycoBlue Mycoplasma Detector assay (Vazyme), according to the manufacturer's instructions.

PD-1/PD-L1 Immune Checkpoint Blockade Assay

PD-1/PD-L1 Blockade Bioassay (Promega) was performed to verify the blocking of PD-1/PD-L1 interaction *in vitro* by selected small-molecule inhibitors. CHO/TCRAct/PD-L1 cells were seeded on 96-well white opaque plates at the density of 15,000 cells and in the volume of 100 µL per well in a growing medium (RPMI-1640 & 10% FBS). Jurkat-EC cells medium was changed for 24 hours, to RPMI-1640 with 1% FBS. On the following day, the 4-fold dilutions of the inhibitors of PD-L1 were prepared in DMSO (BioShop) and then 1000 times diluted in the assay buffer (RPMI-1640 & 1% FBS). The final concentration of DMSO in all samples was kept at 0.05% (v/v). DMSO was used in the assay as a negative control, the positive control was durvalumab, an anti-hPD-L1 monoclonal antibody (Selleckchem), whose final concentration per well was 5 µg/mL and the activation of Jurkat-ECs with durvalumab was set

at 100% of activation. 17 hours after seeding the cells, the medium was harvested and then 40 µL of either prepared serial dilutions of small-molecule compounds or negative or positive control was added to the wells. In the last step, coculture was prepared by seeding on the plate Jurkat T cells at the density of 20,000 cells and in the volume of 40 µL per well. The coculture was maintained at 37°C and 5% CO^{-}_2 for 6 hours, after which the plates were equilibrated at room temperature for 10 minutes and then incubated with Bio-Glo assay reagent (Promega) 75 µL per well for 20 minutes also at room temperature. Jurkat T cells represent effector cells (ECs), whose activity was determined by measuring luminescence on a Spark microplate reader (Tecan). The data was displayed as % of maximum PD-1/PD-L1 blockade (% of maximum blockade), where 0% means that Jurkat-ECs cells were activated with DMSO, while 100% activation was achieved at a concentration of 5 µg/mL of the reference antibody, which results in maximum PD-1/PD-L1 blockade at this concentration. The data points on the graph represent mean values with standard deviation from 3 independent experiments, each conducted in duplicate. The EC_{50} values, representing half-maximal effective concentrations, were determined by fitting the Hill curve to the experimental data using OriginPro 2024b software (OriginLab) and results were shown using GraphPad Prism version 8.0.1.

Biolog Redox Dye Viability Assay

Jurkat-ECs cells, which had 24 hours earlier been changed to RPMI-1640 culture medium with 1% FBS, were seeded into 96-well transparent plates at 20,000 cells/well in a volume of 40 µL/well. 4-fold serial dilutions of small-molecule PD-1/PD-L1 inhibitors in DMSO were prepared and diluted 1,000-fold in a culture medium (RPMI with 1% FBS). Cells were treated with such prepared compounds at 40 µL/well. The control in the experiment was DMSO. The final concentration of DMSO was 0.05% (v/v). The culture plates were incubated for 48 hours at 37°C with 5% CO_2, after which tetrazolium reagent, Biolog Redox Dye MIX MB (Biolog) was added for 4 hours, obtaining its final concentration of 500 µM/well. Absorbance was measured at 590 nm and 750 nm as a reference, using a Spark microplate reader (Tecan). The data points shown in the graph represent the average survival rate of Jurkat-ECs and the standard deviation, relative to cells incubated with DMSO, from 3 independent experiments, each conducted in duplicate. Results were shown using GraphPad Prism version 8.0.1.

Cell Viability MTT Assay

CHO/TRCAct/PD-L1 cells were seeded into 96-well transparent plates at 15,000 cells/well in culture medium (RPMI-1640 + 10% FBS) at a volume of 100 µL/well. The next day, 4-fold serial dilutions of small-molecule PD-1/PD-L1 inhibitors in DMSO were prepared and diluted 1,000-fold in culture medium (RPMI-1640 + 10% FBS). Cells were treated with such prepared compounds at 40 µL/well, after the medium was drawn off from the wells. The control in the experiment was DMSO. The final concentration of DMSO was kept low and constant between samples and was 0.05% (v/v). The culture plates were incubated for 48 hours at 37°C with 5% CO_2, after which Thiazolyl Blue Tetrazolium Bromide (MTT, Sigma Aldrich) was added for 60 minutes to a final concentration of 0.5 mg/mL. The medium was carefully removed from above the cells and the resulting crystals were dissolved in isopropanol with 40 mM HCl. Absorbance was measured at 570 nm and 650 nm as a reference, using a Spark microplate reader (Tecan Spark 20M). The data was displayed as % of DMSO-treated control. The data

points on the graph represent mean values with standard deviation from 3 independent experiments, each conducted in duplicate. Results were shown using GraphPad Prism version 8.0.1.

Isolation of Peripheral Blood Mononuclear Cells (PBMCs)

Anticoagulant citrate dextrose-A-treated blood from healthy donors was purchased from the Regional Center of Blood Donation and Blood Therapy in Krakow, Poland. PBMCs were isolated from whole blood by density gradient centrifugation using Pancoll human separating solution (PAN-Biotech GmbH). The separated cells were washed and resuspended in RPMI 1640 medium (Biowest) containing 10% FBS (Biowest).

T-Cell Activation (TCA) Assay

CHO-K1/TCRAct/*h*PD-L1, CHO-K1/TCRAct, and CHO-K1 cells were seeded on 24-well plates at a density of 50,000 cells per well. After 24 hours, the supernatant was removed, and dilutions of PD-1/PD-L1 inhibitors that were first prepared in DMSO, and then diluted 1000x in the culture medium (RPMI-1640 with 10% FBS and 2mM L-Glutamine) (500 µl per well) were added. Subsequently, 250,000 PBMCs per well (suspended in the 500 µl of culture medium per well) were added to the wells. The final concentrations of tested compounds were: 0.1 µM, 0.5 µM, and 2.5 µM. The final DMSO concentration was maintained at 0.05% (v/v). In this experiment monoclonal antibody, durvalumab was used as a positive control, which was directly diluted in the culture medium and the final concentration was 5 µg/ml. After 48 hours of coculture, the culture medium containing PBMCs was collected 200 µl to the Eppendorf tubes for cytometric bead array and 800 µl to the 5 ml round tubes for TCA Assay). Additionally, 100 µl of TrypLe Select Enzyme (Gibco) was added to each well, followed by 300 µl of culture media after 3 minutes. The contents of the wells were collected again into tubes dedicated to TCA Assay. The cells were then washed twice with 200 µl per sample of Flow Cytometry Staining Buffer (Invitrogen), followed by centrifugation (200 g, 7 minutes at room temperature). The cell pellet was stained with “antibody mix” in the dark at room temperature for 20 minutes. The antibodies that were used for staining: anti-CD4-FITC (cat. no. 555346, Becton Dickinson Biosciences, BD), anti-CD8-BV510 (cat. no. 563919, BD), anti-CD69-APC (cat. no. 555533, BD), anti-CD25-PE (cat. no. 341011, BD), anti-HLA-DR-PerCP (cat. no. 347402, BD), and anti-PD1-PECy7 (561272, BD).

After staining, two washing steps were prepared and the cells were resuspended in 150 µl of Flow Cytometry Staining Buffer and analyzed using a FACSCanto II cytometer. Data analysis was performed using FlowJo software (BD) and GraphPad. The statistical analysis was performed in GraphPad using a one-way analysis of variance (ANOVA), followed by Tukey’s post hoc test: *$p < 0.05$, **$p < 0.01$, ***$p < 0.001$, ***$p < 0.0001$.

Nuclear magnetic resonance (NMR) binding assay

1H NMR measurements were carried out at 300 K on an ultra-shielded 600 MHz Jeol JNM-ECZ600R spectrometer equipped with the HCN Ultracool helium cryo-probe head (with z-gradient coils). To provide a lock signal, 10% (v/v) D2O was added to the samples. Water suppression was achieved with the WATERGATE sequence. The PD-L1 protein was used at a concentration of 0.15 mM. The spectra were processed with Delta 6.0 software.

Supplementary Figures and Tables

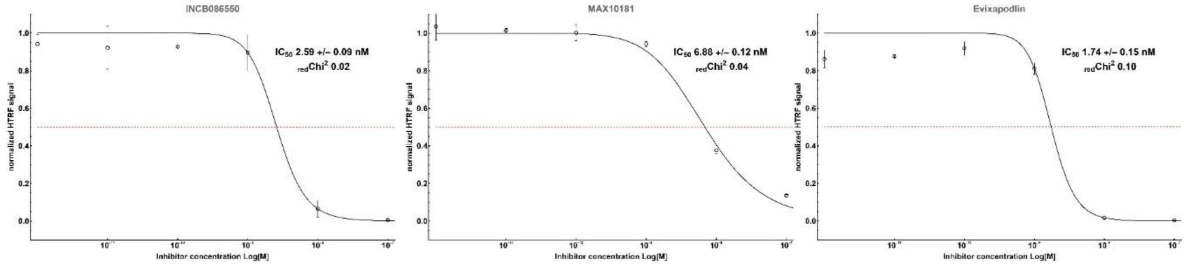

Figure S1. Assessment of the potency of inhibitors INCB086550, MAX-10181, and Evixapodlin in disrupting the human PD-1/PD-L1 complex. Normalized data are represented as dots with SD shown as error bars (n=2). Inhibitor concentrations are displayed on a logarithmic scale. The fitting of Hill's function is shown as a solid line, with the goodness of fit (Chi^2).The half-maximal response is indicated by a dotted red line.

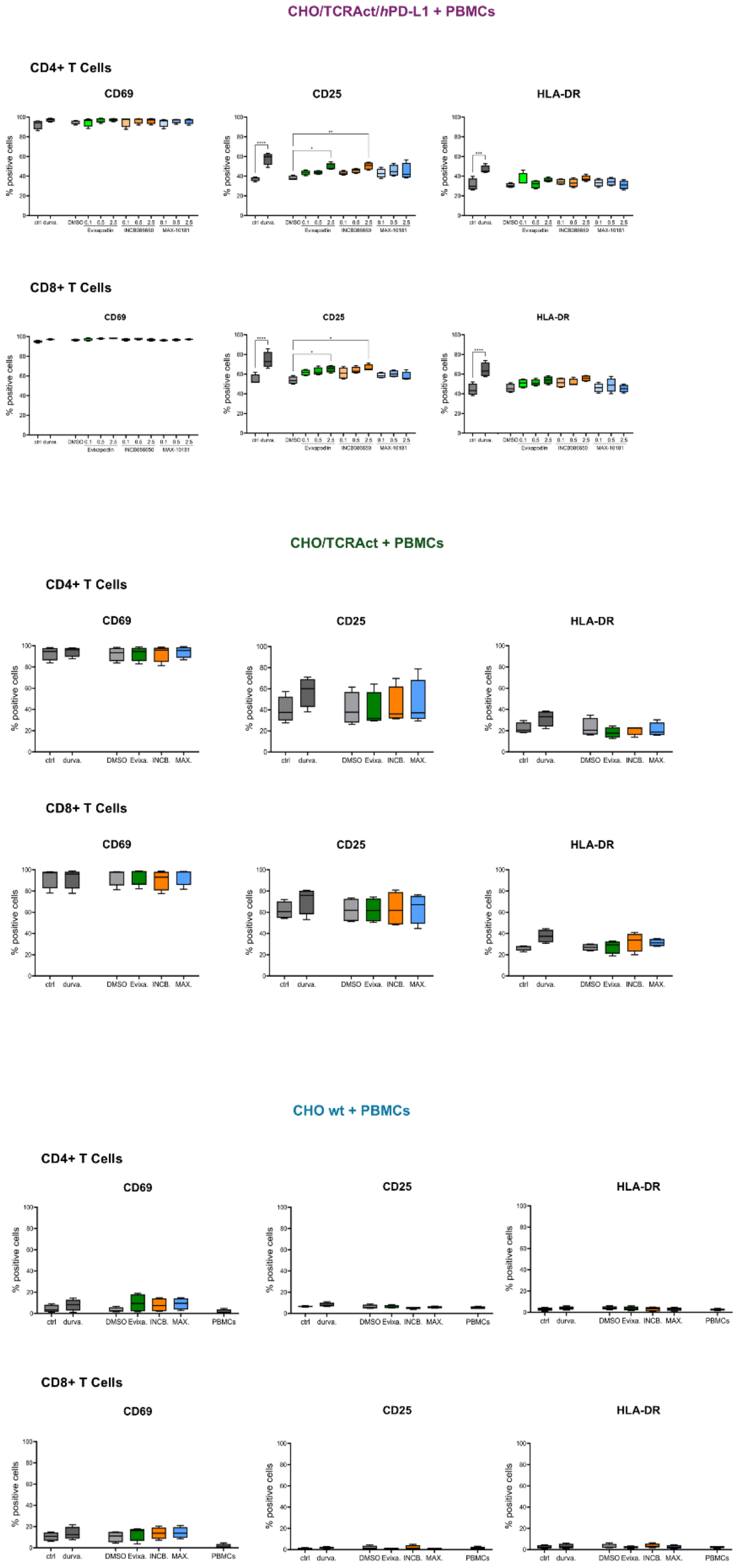

Figure S2. Analysis of the expression of early (CD69) and intermediate (CD25 and HLA-DR) activation markers on human CD4+ and CD8+ T cells. The expression of surface markers was assessed after 48 hours of coculture of PBMCs with CHO/TCRAct/*h*PD-L1, CHO/TCRAct or

CHO-K1 wt in the presence of tested inhibitors at the final concentrations: 0.1; 0.5, 2.5 µM for coculture with CHO/TCRAct/*h*PD-L1, and 2.5 µM for coculture with CHO/TCRAct and CHO-K1 wt, and durvalumab (durva.) at the final concentration 5 µg/ml. On the graphs, the fractions of CD69, CD25, and HLA-DR expressing cells among CD4+ or CD8+ (% positive cells) are shown as calculated data from four independent experiments/donors. Statistical significance was analyzed in GraphPad using one-way ANOVA, followed by the post-hoc Tukey test * p<0.05, ** p<0.01, ***, p<0.000, **** p<0.00001.

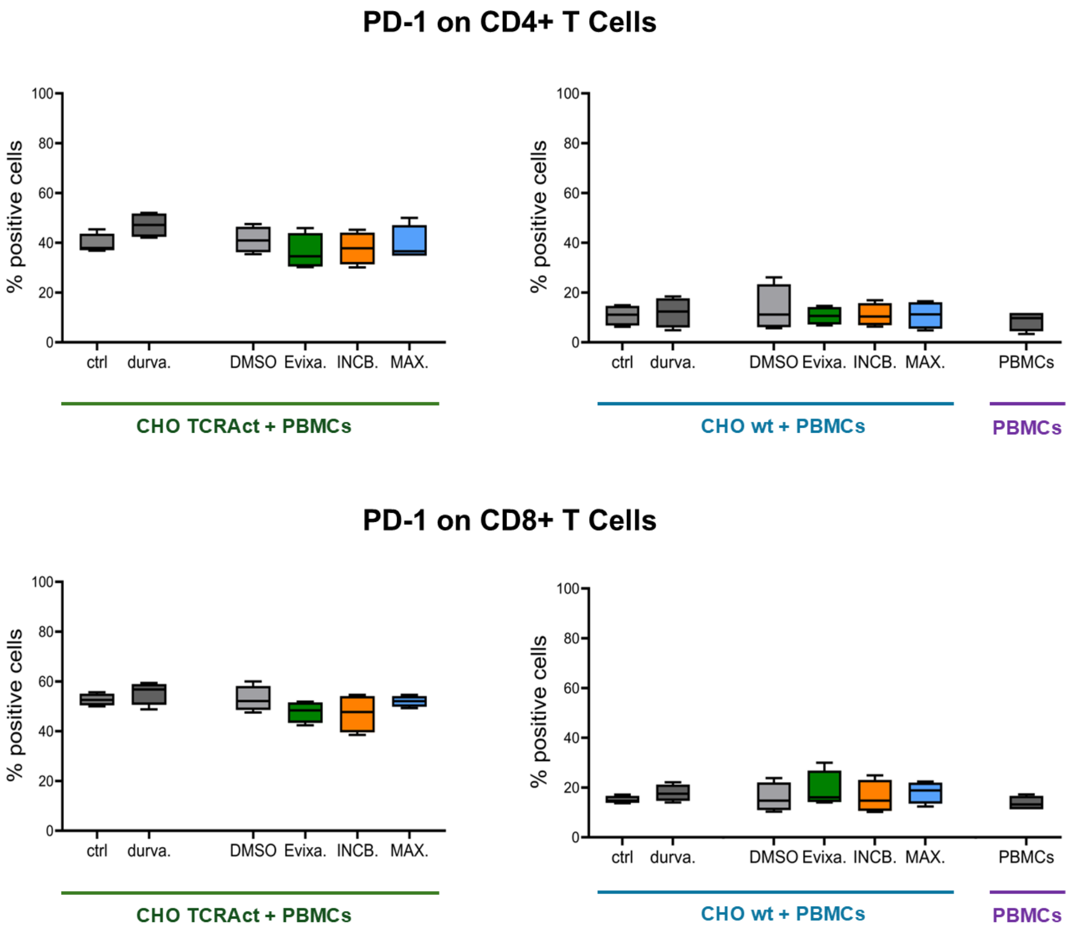

Figure S3. Analysis of the expression of PD-1 as a late activation marker on CD4+ and CD8+ T cells. The expression of surface markers was assessed after 48 hours of co-culture of PBMCs with CHO/TCRAct or CHO-K1 wt in the presence of tested inhibitors at the final concentrations 2.5 µM and durvalumab (durva.) at the final concentration 5 µg/ml. On the graphs, the fractions of PD-1 expressing cells among CD4+ or CD8+ (% positive cells) are shown as calculated data from four independent experiments/donors. Statistical significance was analyzed in GraphPad using one-way ANOVA, followed by the post-hoc Tukey test * p<0.05, ** p<0.01, ***, p<0.000, **** p<0.00001.

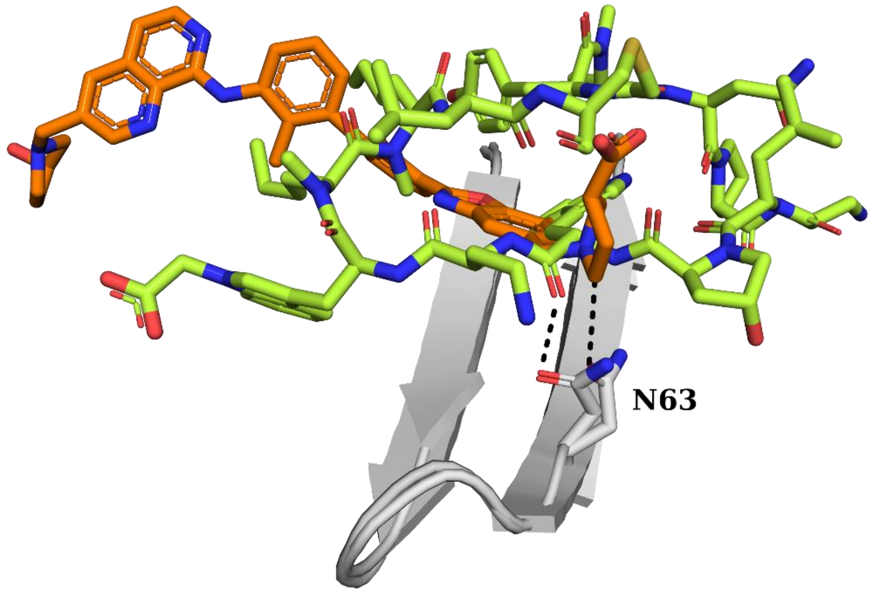

Figure S4. Superposition of X-ray structures of the PD-L1/pAC65 (PDB: 8ALX) and PD-L1/INCB086550 (PDB: 9I0W) complexes. Distal pyridoline-3-carboxylic acid moiety of INCB086550 (orange) is bent when comparing with MAX and Evixapodlin structures (not shown for clarity) to mimic pAC65 (light green) macrocylic peptide contact with Asn63 on the PD-L1 surface.

Table S1. Summary of data collection and refinement statistics. High resolution shell in parentheses.

	**Evixapodlin**	**MAX-10181**	**INCB086550**
PDB code	9I0U	9HRT	9I0W
Data collection			
X-ray source	DESY P11	ESRF ID30A-3	ESRF ID30A-3
Wavelength (Å)	1.033	0.968	0.968
Space group	P 2 21 21	P 2 2 21	P 31 2 1
Cell dimensions			
a, b, c (Å)	52.00, 52.41, 112.02	50.86, 52.03, 111.32	72.07, 72.07, 89.05
α, β, γ (o)	90.00, 90.00, 90.00	90.00, 90.00, 90.00	90.00, 90.00, 120.00
Resolution range (Å)	47.47 - 1.46 (1.49-1.46)	47.14 - 2.30 (2.38-2.30)	62.41 - 2.10 (2.16-2.10)
Rmerge	0.05 (1.36)	0.16 (0.89)	0.20 (1.77)
Rmeas	0.05 (1.45)	0.18 (0.98)	0.22 (1.93)
Rpim	0.01 (0.49)	0.07 (0.41)	0.09 (0.76)
<I/σI>	21.00 (1.50)	6.30 (1.60)	6.00 (1.10)
CC1/2	1.00 (0.68)	0.99 (0.83)	0.99 (0.15)
Completeness (%)	98.90 (88.70)	97.70 (99.30)	94.90 (100.00)
Redundancy / Multiplicity	12.50 (8.10)	5.90 (5.60)	5.90 (6.30)
Total reflections	667878 (19039)	78768 (7301)	89825 (8235)
Unique reflections	53362 (2338)	13321 (1308)	15292 (1312)
Refinement			
Rwork / Rfree	0.16 / 0.20	0.22 / 0.27	0.22 / 0.29
Wilson B-factor	23.40	37.95	37.76
Ramachandran favoured (%)	96.8	96.4	90.8
Ramachandran allowed (%)	2.8	3.6	8.8
Ramachandran outliers (%)	0.4	0	0.4

Author Contributions

J.P. and K.M.-M. designed the research. Methodology: J.P., K.M.-M., J.K.-K, B.M. and L.S. Investigation: A.S., K.G.-M., J.K.-K., B.M., M.St., M.Si., K.W., J.P. Protein expression, purification, crystallization and optimization A.S. and K.G.-M. HTRF experiments K.G.-M. Data refinement A.S., K.G.-M. and J.P. NMR experiments B.M. Cell line experiments and corresponding statistical analysis A.S., J.K.-K., M.St., K.W. Data visualization A.S., J.K.-.K., B.M., K.M.-M. Writing of the original draft A.S., J.K.-K., J.P., K.M.-M. Draft review and editing A.S., J.P., L.S, K.M-M. Funding acquisition, K.M.-M. Supervision J.P., L.S, M.Si., and K.M.-M. All authors discussed the experiments and commented on the manuscript.
